# Calcium oxide, potassium phosphite and a *Trichoderma* enriched compost water suspension protect *Capsicum annuum* against *Phytophthora capsici* by priming the immune system

**DOI:** 10.1002/ps.6401

**Published:** 2021-05-04

**Authors:** Alessio Bellini, Massimo Pugliese, Vladimiro Guarnaccia, Giovanna Roberta Meloni, Lodovica Maria Gullino

**Affiliations:** ^1^ AGROINNOVA – Centre of Competence for the Innovation in the Agro‐Environmental Sector University of Turin Turin Italy; ^2^ Agricultural, Forestry and Food Sciences Department (DISAFA) University of Turin Turin Italy

**Keywords:** pepper, crown rot, systemic acquired resistance, salicylic acid, plant resistance inducers, gene expression

## Abstract

**BACKGROUND:**

Proper management of *Phytophthora capsici* in pepper cultivation is extremely important, since Phytophthora blight is the main disease of this crop worldwide. In the past, the main strategy adopted had been the use of fungicides, causing, in some cases, the development of *P. capsici* resistant strains. In this work three different treatments selected from previous studies (potassium phosphite, calcium oxide and a water suspension from *Trichoderma* sp. TW2 enriched compost) were tested to prove their ability to activate the systemic acquired resistance (SAR) in pepper against *P. capsici*; acibenzolar‐s‐methyl was used as positive control. Two independent growth chamber pot experiments were performed, spatially dividing the site of treatments application (as radical drench) and the site of inoculation (as agar plug on the third leaf).

**RESULTS:**

Leaf lesions were measured, showing a significant reduction on all treated plants compared to the untreated control. To further confirm this hypothesis, the expression levels of three SAR key genes (*CaPBR1*, *CaPO1* and *CaDEF1*) were evaluated though RT‐Real Time PCR at the three end‐point times: T0, T6 and T24. A significant increase of target genes expression at least in one end‐point time in each treated plant was observed. Eventually, statistical overaccumulation of salicylic acid was observed in the upper leaves at the same end‐point times, through HPLC‐MS/MS analysis.

**CONCLUSION:**

This work confirmed the hypothesis that the three treatments tested have the ability to prime the plant immune system, leading pepper to an alert status able to confer a better defence against *P. capsici*. © 2021 The Authors. *Pest Management Science* published by John Wiley & Sons Ltd on behalf of Society of Chemical Industry.

## Graphical abstract

Three treatments (Calcium oxide, potassium phosphite and a *Trichoderma* enriched compost water suspension) were demonstrated to activate the systemic acquired resistance on *Capsicum annuum* plants against *Phytophthora capsici* attacks through the study of genes (*CaPBR1*, *CADEF1* and *CAPO1*) expression and salicylic acid accumulation.

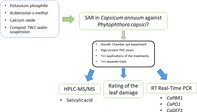

## 1 INTRODUCTION

*Phytophthora capsici* is a filamentous soilborne oomycete considered one of the most disruptive pathogens of some vegetable crops, representing the main problem on pepper (*Capsicum annuum*).^1^ The diseases caused by *P. capsici* depend on the site of infection, ranging on: root, fruit, foliar and crown rot.^2^

Disease management is focused on several management tools including agronomical strategies, chemical fungicides and soil disinfestation.^3^ A good control of the disease was guaranteed with the use of fungicides belonging to phenylamide class (PAF), in particular metalaxyl and its enantiomer mefenoxam. However, the fact these molecules have a specific site of action together with some aspects of *P. capsici* biology, i.e., the sexual recombination through oospores production when two mating types are paired and the mutations in zoospores genome mediated by UV light since sporangia are hyaline,^4^ led to the selection of some resistant strains. On the other hand, pepper plant breeding is promising but not resolutive: resistant varieties have often worse horticultural characteristics.^5^ Moreover, it has been proved that genetic resistance might be effective only against some specific strains of *P. capsici*.^6^ For these reasons, integrated management of Phytophthora blight is highly recommended. Beyond the agronomical practices, such as increasing water draining, decreasing watering if not necessary and destroying infected plants, further control strategies need to be developed and implemented in practice. Among them one interesting technique is the employ of the so‐called Plant Resistance Inducers (PRIs): inorganic salts, microorganisms and biomolecules able to prime the plant immune system guaranteeing a more efficient and quicker response to pathogens's attack.^7^ PRIs, acting on the host plants rather than targeting directly the pathogen, have several advantages: (i) they are considered safer for both environment and human health compared to chemical fungicides, (ii) they can be used on several pathosystems, and (ii) they are less able to induce resistance in target pathogens.^8^ More specifically, PRIs act triggering two different molecular pathways through different stimuli: the systemic acquired resistance (SAR) and the induced systemic resistance (ISR). SAR is salicylic acid dependent, it can be activated by both biotic and abiotic factors and it is able to prime the plant immune system against biotrophic and hemibiotrophic pathogens.^9^ ISR is regulated by jasmonic acid and ethylene, it is generally activated by symbiosis with bacteria and fungi and it works against necrotrophic pathogens.^10^ The activation of salicylic acid dependent pathway leads to overexpression of genes encoding defence proteins such as: the pathogenesis related proteins,^11^ the defensins^12^ and the peroxidases.^13^ These proteins exert a concerted action against infections that guarantee a higher level of protection against plant pathogens. Based on previous experiments showing treatments able to reduce the disease severity and incidence on a wide range of pathosystems,^14^, ^15^, ^16^, ^17^ two mineral salts (potassium phosphite and calcium oxide) and a compost enriched with a *Trichoderma* strain,^18^ were selected to understand their mode of action; acibenzolar‐s‐methyl was used as a positive control.^19^ Potassium phosphite has already been proved to be effective in stimulating the SAR in model species like *Arabidopsis* and potato.^20^, ^21^ Several compost treatments were demonstrated to enhance the immune systems of plants against different pathogens.^22^, ^23^ Moreover, *Trichoderma* spp. were demonstrated as able to activate the systemic acquired resistance pathway.^24^, ^25^ *Trichoderma* sp. TW2 enriched compost has been used to prevent *P. capsici* infection against summer squash^26^; its mode of action was related with the alteration of rhizosphere mycobiota, but induction of resistance was also hypothesized. Calcium oxide was demonstrated to be effective in different pathosystems,^14^, ^15^ similarly, it is known that calcium ions are involved in the SAR pathway.^27^, ^28^ The aim of this work was to demonstrate the ability of the treatments chosen to act as PRIs in *C. annuum* against *P. capsici*. The investigation was conducted by studying: (i) the expression level of three genes representing the most studied and important families of SAR related genes (*CaPO1* –peroxidases, *CaPBR1 –* pathogenesis related proteins and *CaDEF1 ‐* defensins)^29^, ^30^ through RT‐Real‐Time PCR assays, and (ii) the accumulation rate of salicylic acid through HPLC‐MS/MS analysis.

## 2 MATERIALS AND METHODS

### 2.1 Setting of the experiments

The experiment was conducted using 40 plants for each treatment. Twenty plants for each treatment were used to sample the leaves for chemical and molecular analysis, while the other 20 plants were used for the assessment at T48 of mycelium growth, which was performed measuring the expansion of the lesion. The experiment was carried out twice independently.

### 2.2 Statistical analyses

The homogeneity of the data (Levene's test) allowed to unify the two trials. Statistical analyses were performed using the SPSS software (IBM SPSS Statistics, Westland, MI, USA). ANOVA and Duncan *post hoc* tests were performed to establish the statistical values of the differences (*P* < 0.05) for all data collected (damage on the leaf, molecular and chemical analyses).

### 2.3 Plant material and inoculum

*Capsicum annuum* seeds were sown in a peat substrate (Tecno 2, 70% white peat and 30% clay, pH 5.5–6, N 110–190 mg L^−1^, P_2_O_5_ 140–230 mg L^−1^, K_2_O 170–280 mg L^−1^, Turco Silvestro Terricci, Bastia d'Albenga, SV, Italy) and left in a growth chamber at 24 ± 1 °C for 3 weeks. After that seedlings were transplanted with one plant for every pot (7 × 7 cm) filled with the same peat used for the germination. A strain of *P. capsici* from the Agroinnova collection (1/63) was grown at 24 ± 1 °C in corn meal agar plates for 1 week before the inoculation.

### 2.4 Treatments and inoculation

Potassium phosphite, calcium oxide and acibenzolar‐s‐methyl were prepared dissolving commercial products in deionized water as in Table 1. Compost water suspension (Compost w.s.) was prepared in flasks with 50% of green compost fortified with *Trichoderma* sp. TW2 (ANT's Compost M, AgriNewTech srl) and 50% of deionized water kept to stir overnight. After that, the supernatant was filtered with paper to remove the compost. The compost used was the same already applied in previous trials.^23^, ^31^ Treatments were applied as radical drench with an amount of 100 mL per each pot, two times: 72 and 24 h before inoculation with the pathogen; the not inoculated and the untreated control plants were drenched with water. The inoculation of *P. capsici* was carried out after 24 h from the last PRIs treatment, briefly: a 5 mm (diameter) plug disk of fresh mycelium was cut from Corn Meal Agar plates and placed on the third leaf previously moisturized with sprayed water. After the inoculation, plants were covered with plastic bags to create a moist chamber for 24 h.

**Table 1:** List and amount of the treatments used in the trials

Treatment	Commercial name	Dosage (a. i.) mL or g L^−1^	% Active ingredient
Potassium phosphite P_2_O_5_ 52%, K_2_O 42%	Alexin	1.03 + 1.05 g L^−1^	52 + 42
*Acibenzolar‐s‐methyl*	Bion	0.0125 g L^−1^	50
*Calcium oxide*	Califol	1.10 mL L^−1^	22.1
*Compost water suspension*	ANT's Compost M	50% compost 50% water	—
*Untreated inoculated control*	—	—	
*Untreated not inoculated control*	—	—	

### 2.5 Analyses of target gene expression

The third leaf of inoculated and not inoculated plants was collected in biological triplicate at three end points: immediately before inoculation (T0), 6 h after inoculation (T6) and 24 h after inoculation (T24). The leaves were frozen with liquid nitrogen and stocked at −80 °C until the extraction. 50 mg of each sample was grinded in liquid nitrogen with pestles and mortars previously sterilized for 3 h at 170 °C to eliminate any nuclease. The samples were then extracted using the RNase‐Free DNase I Kit (Norgen Biotek Corp., Canada) following the manufacturer's instructions. RNA was quantified using a NanoDrop 2000 spectrophotometer (Thermo Fisher Scientific, Waltham, MA, USA). DNA contamination was eliminated using the TURBO DNA‐free™ Kit (Thermo Fisher Scientific, Waltham, MA, USA) following the rigorous treatment as in the manufacturer's instructions. The samples were retrotrascribed with High‐Capacity cDNA Reverse Transcription Kit (Thermo Fisher Scientific, Waltham, MA, USA). The obtained cDNA was analyzed in biological and technical triplicate using a StepOne‐PlusTM Real‐Time System with the Power SYBR™ Green PCR Master Mix (Applied Biosystems, Foster City, CA, USA). Three key genes of the systemic acquired resistance were analysed to assess their expression levels: *CaPBR1*, *CaPO1* and *CaDEF1*, while *CaActin* (the actin gene) was used as housekeeping gene. The primer^32^ list and the RT Real‐Time PCR conditions are described in Table 2. The expression levels of the target genes were calculated through 2^−ΔΔCT^ method.

**Table 2:** Description of primer sets and amplification conditions of RT Real‐Time PCR assays

Gene	Primers	Real time PCR conditions
Pathogenesis related protein (PR)1 ‐ *CaBPR1*	CAPR1‐F	40 Cycles
	CAPR1‐R	95° 10′′, 56° 30′′, 72° 30′′
Defensin ‐ CaDEF1	CADEF‐F	40 Cycles
	CADEF‐R	95° 10′′, 56° 30′′, 72° 30′′
Peroxidase ‐ CaPO1	CAPO1‐F	40 Cycles
	CAPO1‐R	95° 10′′, 56° 30′′, 72° 30′′
Actin ‐ CActin2	CACTIN2‐F	40 Cycles
	CATIN2‐R	95° 10′′, 56° 30′′, 72° 30′′

All primers and amplification conditions were taken from Zhang et al.,^32^

### 2.6 Chemicals and reagents

HPLC‐grade acetonitrile, methanol, acetic acid and formic acid were purchased from VWR International (Radnor PA, USA) and Sigma‐Aldrich (St Louis, MO, USA). Water was obtained using a Milli‐Q water purification system (G. Maina, Pecetto Torinese, Italy). The standard compound salicylic acid (purity ≥99%) was supplied by Sigma‐Aldrich (St Louis, MO, USA). Two stock standard hormone solutions were made in methanol at the concentration of 1000 and 10 μg mL^−1^, respectively, and both stored in the dark at −20 °C, while a standard working solution was prepared daily by diluting the standard stock solution to obtain calibration curve.

### 2.7 Extraction and HPLC‐MS/MS analysis of salicylic acid

Phytohormone analysis from pepper leaves was performed following the procedure previously reported by Pagliarani et al.,^31^ with minor modifications. Pepper leaves samples were frozen in liquid nitrogen and homogenized with mortars and pestles. About 0.2 g of sample was accurately weighed, transferred to 2 mL centrifuge tube and dissolved in 2 mL of extract solution (methanol: water, 80:20, v/v and acidified with 0.1% acetic acid). The solution was shaken at 4 °C overnight in the dark and filtered with a 0.2 μm cellulose filter. Finally, the supernatant was analyzed by HPLC‐MS/MS. Quantification of salicylic acid was performed using a 1260 Agilent Technologies system consisting of a binary pump and a vacuum degasser, connected to a Varian autosampler, Model 410 Prostar (Hansen Way, CA, USA), equipped with a 20 μL loop coupled to a Varian 310‐MS TQ Mass Spectrometer. A Luna 3 μm phenyl‐hexyl (150 × 2 mm, Phenomenex, Torrance, CA, USA) under a flow of 200 μL min^−1^, was used for the chromatographic separation. Solvent A was H_2_O, while solvent B was CH_3_CN, both acidified with 0.1% formic acid. The gradient elution was programmed as follows: 0–7 min isocratic 40% solvent B, followed by a linear gradient from 40% to 100% B in 5 min and holding at 100% B for 4 min. The injection volume was 10 μL and the mass spectrometer was operated in the ESI (electrospray) positive ionization mode using multiple reaction monitoring (MRM) mode. The quantification ion transitions selected was 137 > 93 (16 eV). The collision gas (Ar) pressure was set at 2 mbar for all experiments.

## 3 RESULTS

### 3.1 Containment of *P. capsici* growth

**Figure 1:**
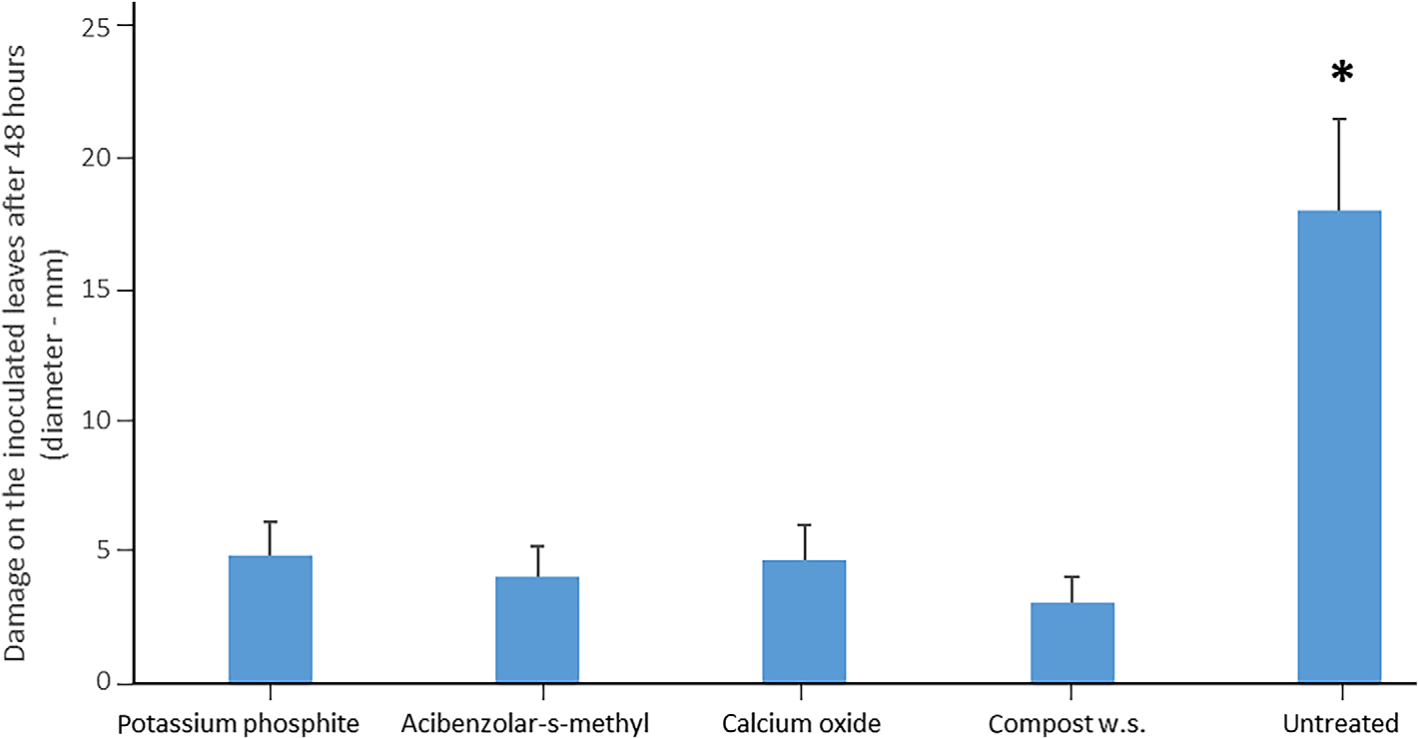
Containment of *P. capsici* growth in the inoculated leaves compared assessed as diameter (cm) of the damage after 48 h post inoculation. The asterisk specifies the significative difference obtained with On‐way Anova followed by the Duncan post‐hoc test.

The symptoms on the leaves were assessed 48 h after the inoculation, measuring the diameter of the oomycete growth (mm). All treated plants showed a statistical reduction of the oomycete growth (Fig. 1) compared to the one recorded on the leaves of untreated plants. Average values of 3, 4, 4.6 and 4.8 mm were observed in compost water suspension, acibenzolar‐s‐methyl, potassium phosphite and calcium oxide treated plants, respectively, compared to an average value of 18 mm measured on the leaves of untreated plants.

### 3.2 Chemical analyses

**Figure 2:**
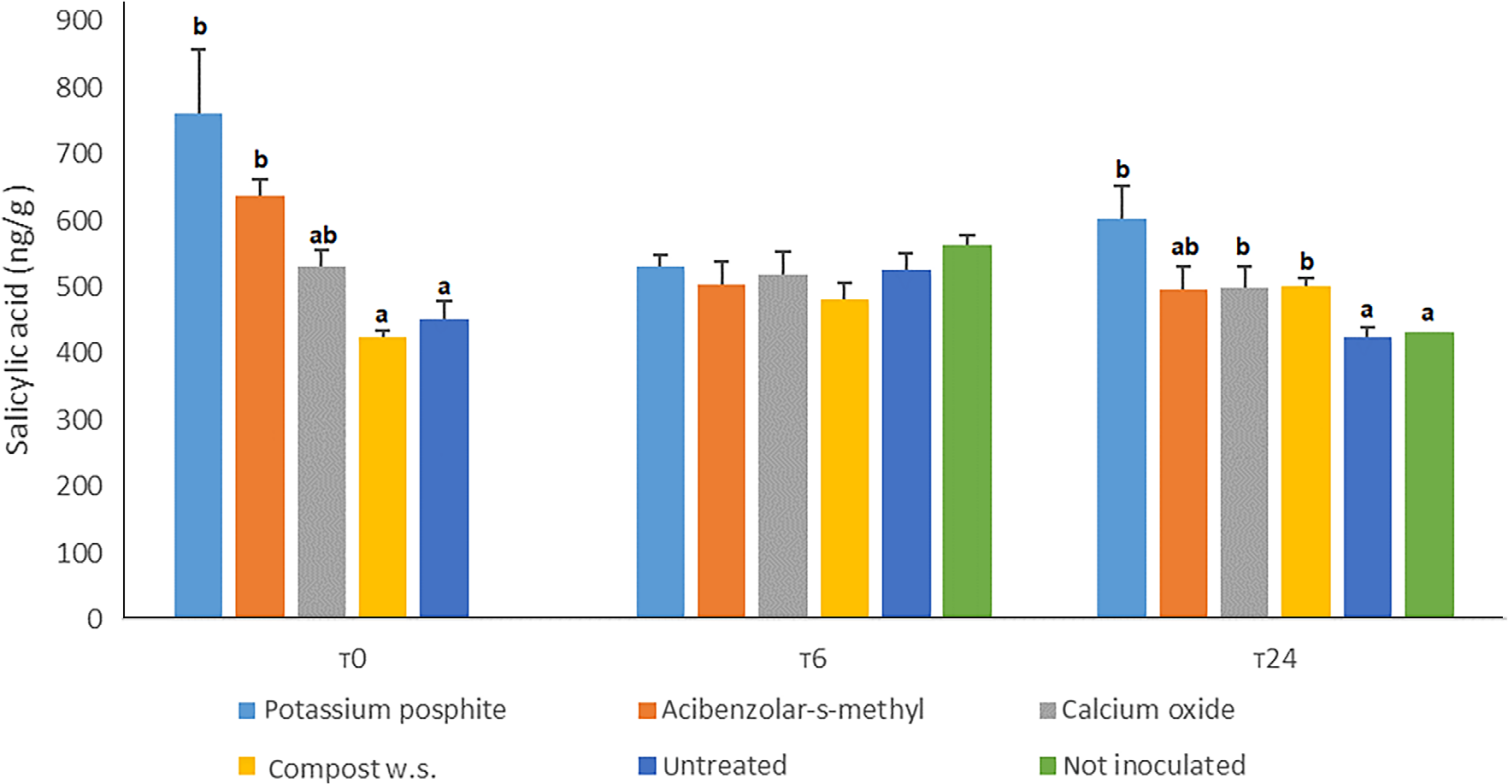
Accumulation of salicylic acid in the upper leaves at T0, T6 and T24 (post accumulation). Data were evaluated by HPLC‐MS/MS analysis. Letters refers to Duncan post‐hoc test performed after one way Anova. Data from first and second replication are here condensed.

At T0, potassium phosphite and acibenzolar‐s‐methyl treated plants showed statistical overaccumulation of salicylic acid compared to untreated ones (respectively 760.2, 684.4 and 452.6 ng g^−1^), while calcium oxide (548.6 ng g^−1^) and compost suspension (431.7 ng g^−1^) treated plants did not statistically differentiate from the control. At T6, no plant showed statistical overaccumulation compared to the control, ranging from a minimum of 482 ng g^−1^ (compost treated) and a maximum of 563.3 ng g^−1^ (not inoculated untreated control). At T24, potassium phosphite, calcium oxide and compost water suspension treated plants (603.1, 528.5 and 521.6 ng g^−1^) overaccumulated salicylic acid compared to acibenzolar‐s‐methyl (497.2 ng g^−1^) and the untreated (413.9 ng g^−1^) and not inoculated (399.9 ng g^−1^) controls. Data shown in Fig. 2.

### 3.3 Analyses of target gene expression

**Figure 3:**
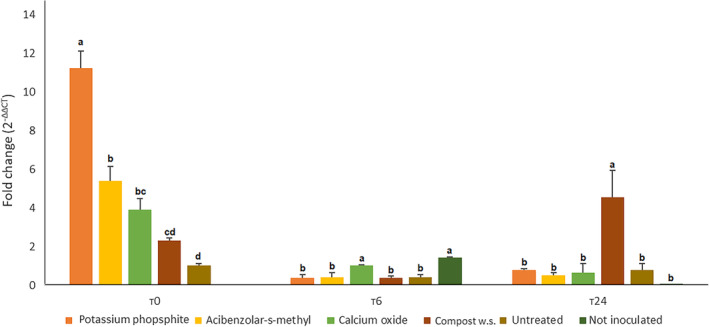
Expression levels of *CapPBR1* gene in inoculated leaves (third) at T0, T6 and T24 (hours post inoculation). The data were calculated with RT‐Real‐Time PCR assay (*CaActin* gene was used as housekeeping) and extrapolated through 2^−ΔΔCT^ method. Letters refers to Duncan post‐hoc test performed after one way Anova. Data from first and second replication are here condensed.

**Figure 4:**
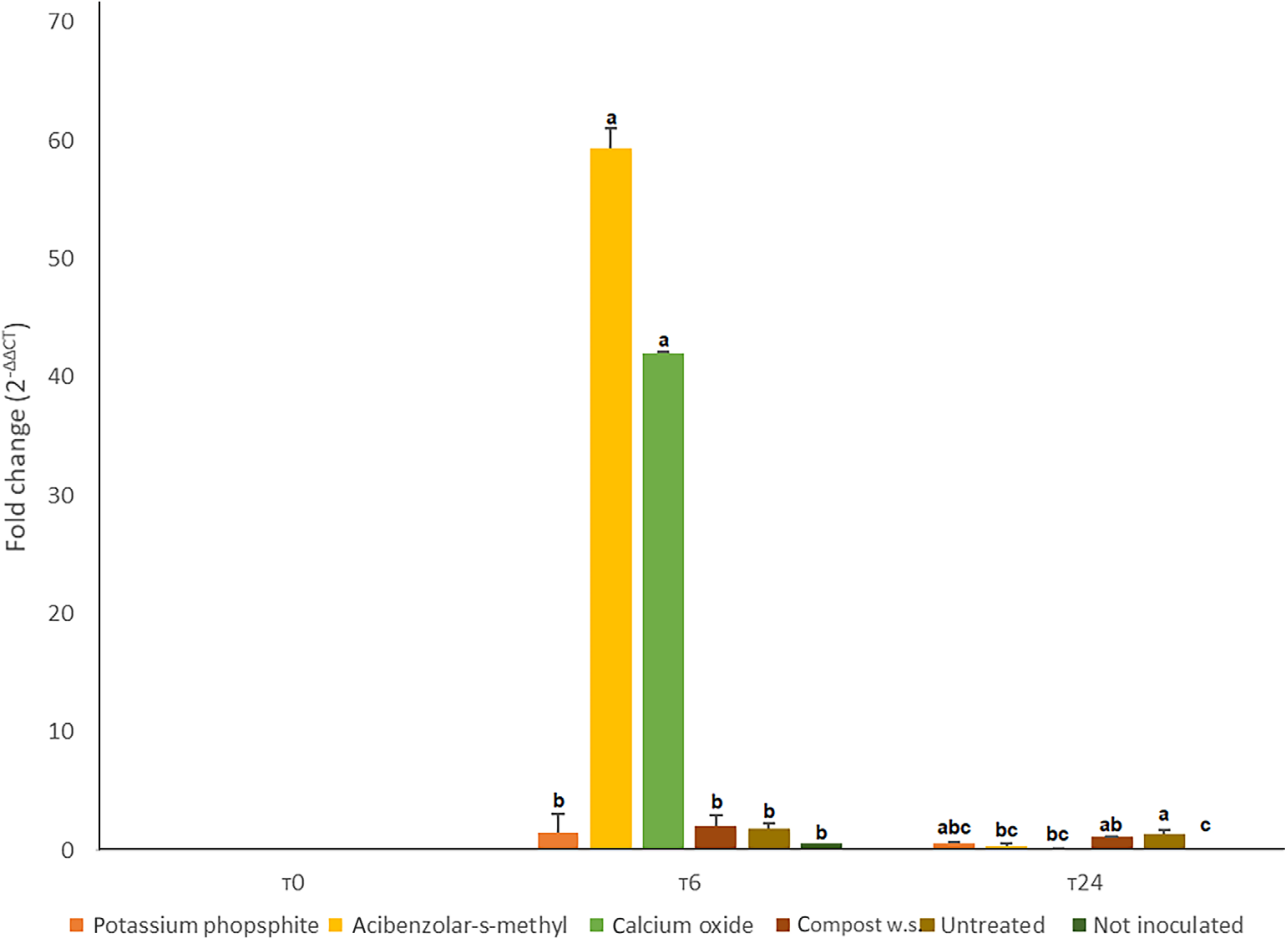
Expression levels of *CaPO1* gene in inoculated leaves (third) at T0, T6 and T24 (hours post inoculation). The data were calculated with RT‐Real‐Time PCR assay (*CaActin* gene was used as housekeeping) and extrapolated through 2^−ΔΔCT^ method. Letters refers to Duncan post‐hoc test performed after one way Anova. Data from first and second replication are here condensed.

**Figure 5:**
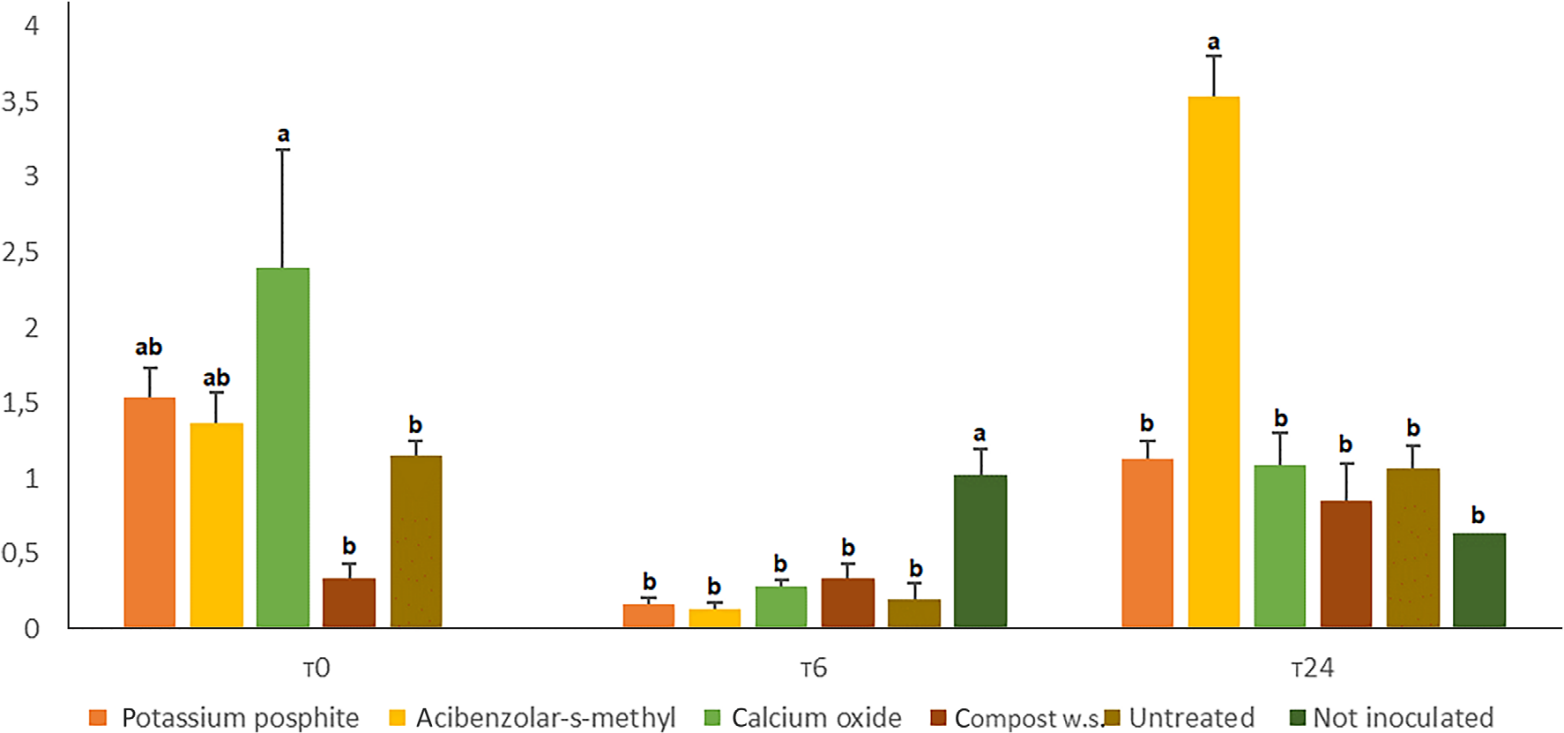
Expression levels of *CaDEF1* gene in inoculated leaves (third) at T0, T6 and T24 (hours post inoculation). The data were calculated with RT‐Real‐Time PCR assay (*CaActin* gene was used as housekeeping) and extrapolated through 2^−ΔΔCT^ method. Letters refers to Duncan post‐hoc test performed after one way Anova. Data from first and second replication are here condensed.

*CaBPR1* gene was generally more expressed at T0: potassium phosphite, acibenzolar‐s‐methyl and calcium oxide treated plants showed overexpression compared to the untreated control (respectively 8, 5, and 4 times). At T6 calcium oxide treated plants showed no statistical difference compared to the untreated not inoculated control. All of the others showed a significant reduction of *CaBPR1* gene expression. At T24, *CaBPR1* gene was overexpressed in compost water suspension treated plants, while all the other plants did not statically differentiate. Peroxidase gene was not expressed at T0 in any sample; while at T6 a great overexpression of *CaPO1* gene was observed in samples from acibenzolar‐s‐methyl and calcium oxide treated plants (49 and 26×) compared to the untreated control. At T24 no differences were observed. *CaDEF1* gene was overexpressed in calcium oxide treated plants at T0; whereas at T6 there was a downregulation compared to the untreated not inoculated control. At T24 acibenzolar‐s‐methyl treated plants showed overexpression of *CaDEF1* compared to the controls. Data were collected in Figs 3, 4, 5, respectively for *CaPBR1*, *CaPO1* and *CaDEF1*.

## 4 DISCUSSION

The adoption of proper management strategies against *P. capsici* has a key role for *C. annuum* cultivation both in open field and in greenhouse, since this pathogen represents the major cause of significant economic losses. The aim of this study was to elucidate the mechanisms of action of the treatments here considered (potassium phosphite, calcium oxide and a water suspension from a *Trichoderma* enriched compost) in the specific pathosystem *P. capsici* – *C. annuum*. To avoid a possible direct biocide effect caused by the treatments, the site of inoculation and the site of treatments were spatially divided, the first one was performed on the third leaf, while the treatments were given as radical drench as in Zhang et al.^32^ The disease severity on the inoculated leaves was calculated as diameter of the lesion caused by *P. capsici*'s growth 48 h after the inoculation. The treated plants showed a significant statistical reduction of the lesions compared to the untreated ones, which could be determined by a range of several factors.

To better investigate if, among these factors the activation of systemic acquired resistance was involved, both chemical and molecular analyses were conducted on tissues sampled at three endpoint times (T0, T6 and T24). RT Real‐Time PCR assay was used to evaluate the expression levels of *CaBPR1, CaPO1 and CaDEF1* on the third leaves. HPLC‐MS/MS analysis was performed on upper leaves samples to assess the accumulation of salicylic acid in the tissues above the ones colonized by the pathogen, since it is known that the hormone generally has an acropetal movement inside the plants vascular system.^33^ At T0 (72 and 24 h after the treatments) both overaccumulation of salicylic acid (in potassium phosphite and acibenzolar‐s‐methyl treated plants) and overexpression of *CaBPR1* and *CaDEF1* (in potassium phosphite and calcium oxide treated plants) were demonstrated.

Genes expressing pathogenesis related proteins are highly inducible by the accumulation of salicylic acid^34^ and have been proved to be overexpressed in potato plants 72 h after the treatments with potassium phosphite compared to control.^35^ Defensins are proteins that exert a great antifungal activity^36^ helping the plants to cope with infections. *CaPO1* gene was not expressed at T0 in accordance with the literature, since it is highly stress inducible.^37^ The fact that potassium phosphite and calcium oxide treatments determined an overaccumulation of salicylic acid and/or overexpression of *CaBPR1* and *CaDEF1* before the inoculation, highlights a clear priming effect, suggesting that the induction of resistance in *C. annuum* by these treatments could be helpful not only against *P. capsici* infection, but also against other biotrophic and hemibiotrophic pathogens. At T6 no overaccumulation of salicylic acid was observed in the upper leaves, in accordance with Rasmussen et al.,^38^ where it was demonstrated that at least 4–6 h are needed to synthetize the salicylic acid signal in the infected tissues and then translocate it.

Acibenzolar‐s‐methyl and calcium oxide treated plants showed a great over expression of *CaPO1* gene at T6, as already proved with other treatments by Wang et al.,^39^ confirming the involvement of these treatments in the activation of SAR pathway leading to a more efficient oxidative burst compared to the untreated plants. At T24, overaccumulation of salicylic acid was detected in potassium phosphite, calcium oxide and water compost treated plants. While acibenzolar‐s‐methyl treatment was able to overexpress *CaDEF1* gene in accordance with Zhang et al.,^32^ where *P. capsici* induced an overexpression of the defensin gene in pepper 24 h after the inoculation. Moreover, compost water suspension treated plants overexpressed *CaPBR1* gene at T24 in accordance with Asghari et al.,^40^ and Esmail et al.,^41^ who demonstrated overexpression of the target gene 24 h after inoculation respectively in: grapevine plants against *Agrobacterium tumefaciens* and in *Triticum aestivum* plants primed with diverse *Trichoderma* strains against *Puccinia graminis* f. sp*. tritici*. The fact that at T24 overexpression of target genes and overaccumulation of salicylic acid were here demonstrated, suggests that the immune system of the treated plants was still in an alert status.

No overexpression of *CaPO1* gene was detected at T24, in accordance with Do et al.,^13^ These results confirmed the ability of the treatment here considered to act as PRIs, enhancing *C. annuum* defences against *P. capsici*. The overexpression of the three target genes in inoculated leaves of treated plants proved that the molecular pathway was activated in a more efficient way, furthermore the overaccumulation of salicylic acid in upper leaves corroborated the hypothesis. The method used to prove this stimulation of the plant immune system was effective since it allowed to study the systemic response from the roots, passing by the inoculated leaf to the upper ones.

## 5 CONCLUSION

In conclusion these findings highlight the priming effect of the treatments considered in this study, thus suggesting that the mode of action is not, at least only, related with the direct interaction with the pathogen. To the best of the knowledge there are not similar studies on this pathosystem which consider the treatments used in this research analyzing both the expression of resistance genes and the accumulation of salicylic acid. More research will be undertaken to test if their ability to induce the SAR is maintained in field conditions and also in other pathosystems.

## CONFLICT OF INTEREST

Massimo Pugliese declares he has a financial interest as he is a shareholder in the AgriNewTech company that provided some of the products tested in this article. The remaining authors declare that the research was conducted in the absence of any commercial or financial relationships that could be construed as a potential conflict of interest.
